# Is entropy objective? Rethinking the foundations of irreversibility and the second law

**DOI:** 10.1093/pnasnexus/pgag288

**Published:** 2026-08-26

**Authors:** Juan M R Parrondo

**Affiliations:** Departamento de Estructura de la Materia, Física Térmica y Electrónica and GISC, Universidad Complutense de Madrid, Madrid 28040, Spain

**Keywords:** entropy, irreversibility, stochastic thermodynamics, ergotropy

## Abstract

The standard formulations of statistical mechanics and the explanations of irreversibility rely on the way we describe physical systems. They use concepts such as coarse-graining or macroscopic states, which depend on the observables used to describe the system and their precision. In this Perspective, we examine the extent to which entropy, irreversibility, and the second law of thermodynamics can be regarded as observer-independent. To this end, we use Einstein’s observable-dependent entropy and discuss the objectivity of several concepts and phenomena separately: irreversibility, equilibrium, entropy, temperature, and the second law. We also discuss the role of symmetry-breaking transitions in generating low-entropy states and in the arrow of time, as manifested through traces and footprints. Finally, we discuss the mechanical rationale of the second law based on ergotropy and passive states.

## Introduction

Two identical gases, labeled 1 and 2, are confined in boxes of equal volume. The two gases have the same density, and their internal energies are $E_{1}$ and $E_{2}$, with $E_{1}>E_{2}$. If the boxes are brought into contact and the gases are allowed to exchange energy, the compound system evolves in a familiar way: energy flows from gas 1 to gas 2 until both reach the same energy, up to fluctuations of order $1/\sqrt{N}$, where *N* is the number of molecules in each gas.

This is an irreversible phenomenon and is entirely objective. As pointed out by Albert (1, 2), it does not depend on the information accessible to a macroscopic observer, on coarse-graining, on our ignorance of the microscopic state of the gases, or on any other observer-dependent feature. A large number of molecules is required to observe irreversibility clearly, however. If $(E_{1}-E_{2})/E_{1}≃1/\sqrt{N}$, then the behavior of the two gases will not exhibit any clear arrow of time. Irreversibility is always masked by fluctuations, yet it remains an objective fact of nature.

Despite all this, irreversibility is typically regarded as an emergent property at large scales. The main reason is that, for instance, if we zoom in on a gas undergoing an irreversible expansion, we observe molecules moving and colliding in accordance with Newton’s laws, which are invariant under time reversal. Reconciling this reversible behavior at the microscale with macroscopic irreversibility is one of the fundamental problems of statistical mechanics.

Boltzmann proposed a solution that is considered satisfactory by a majority of physicists (3). It rests on the idea that irreversibility arises from the way macroscopic observers describe physical systems. Boltzmann’s argument is well known: he introduces macrostates, each compatible with many microstates, defines the entropy of a macrostate as the logarithm of the number of compatible microstates, and concludes that an ergodic system evolves irreversibly toward macrostates of higher entropy.

This reasoning involves an element of arbitrariness in the definition of macrostates. As Lebowitz acknowledges (3), “while this specification of the macroscopic state clearly contains some arbitrariness, this need not concern us too much, since all the statements we are going to make about the evolution of [a macrostate] *M* are independent of its precise definition as long as there is a large separation between the macro- and microscales.” Yet, this arbitrariness raises legitimate questions, particularly in light of developments in the control and manipulation of microscopic and nanoscopic systems in contact with thermal baths. Does entropy depend on the specification of macroscopic states if we include mesoscopic or microscopic observables? Can the notion of a “large separation between the macro- and microscales” be formulated in a more precise way?

In this Perspective, I examine to what extent the subjective elements in thermodynamics and statistical mechanics can be eliminated. The central idea is that Boltzmann’s argument can be made entirely objective by following a strategy that goes back to Einstein (4): defining an entropy associated with observables rather than macrostates. Within this framework, and under the ergodic hypothesis, one can prove that the irreversible behavior of certain observables is objective. Earman presents a similar argument in Ref. (2), claiming that the increase of Boltzmann entropy is objective, *given the appropriate coarse-graining*, i.e. it is a fact of nature that the coarse-grained description exhibits an irreversible behavior. This idea is also implicit in Ref. (5), an exhaustive study of objectivity in thermodynamics and statistical mechanics.

The consequences of this observable-dependent notion of entropy, however, have not been thoroughly explored. This work aims to analyze them in detail. Along the way, we will encounter surprising situations that challenge standard intuitions. For example, we discuss a case in which a microscopic observable exhibits irreversible behavior while the system’s macroscopic state evolves reversibly. We will also be forced to separately analyze the objectivity of concepts and principles that are usually treated together: irreversibility, equilibrium, entropy, temperature, and the second law.

The resulting picture of irreversibility largely agrees with the standard one: unlikely initial conditions relax toward the most probable values of certain observables. A crucial question then arises: how are such low-entropy initial conditions generated? I will introduce a mechanism for their origin based on symmetry breaking in an expanding universe. Symmetry breaking can also play a role in the formation of traces and footprints, phenomena that reflect the presence of an arrow of time and whose relationship with the increase of entropy remains controversial (2, 6–9).

The second law requires a separate discussion, since it concerns the possibility of extracting energy from a system through a cyclic process and therefore applies to systems driven by an external agent. Maxwell already realized that the ability of such an agent to extract energy depends on the information available about the state of the system. He illustrated this idea with his celebrated demon, which has been discussed for more than a century (10, 11). The standard strategy for removing the elements of subjectivity introduced by the Maxwell demon is to treat the demon itself as a physical system and account for the energetic costs associated with its operations (11). This has been successfully achieved in many contexts, reducing the various versions of Maxwell’s demon to systems operated by external agents with limited information about the system’s state (nonfeedback protocols). However, the question of whether it is possible or not to extract energy from a system remains. We address this question using the concepts of passive systems (12, 13) and ergotropy (14).

Finally, a disclaimer. This article does not aim to provide a complete and consistent formulation of thermodynamics and statistical mechanics. This would require solving *all* the foundational problems of these disciplines. Instead, it focuses on the problem of objectivity versus subjectivity of irreversibility and the second law. In particular, I will assume ergodicity in Boltzmann’s sense, i.e. that systems explore their accessible microstates uniformly. The implications and limitations of this assumption, as well as the role of ergodicity and mixing in statistical mechanics, have been extensively discussed elsewhere (15–19).

The discussion will be circumscribed to classical systems. Questions concerning objectivity in quantum mechanics pose additional challenges even for systems with few degrees of freedom. By contrast, classical mechanics provides an observer-independent description of physical systems, making it a natural framework for examining whether entropy should itself be regarded as objective or observer-dependent. Insights from classical analysis may also illuminate similar questions in quantum physics.

## Boltzmann’s explanation of irreversibility

In this section, I outline the modern version of Boltzmann’s argument (3). Let *Γ* denote the phase space—the set of all microscopic states—of a system. Each point x=(q,p) in *Γ* corresponds to all the coordinates $q\equiv (q_{1},\ldots ,q_{M})$ and conjugate momenta $p\equiv (p_{1},\ldots ,p_{M})$ of the *M* degrees of freedom of the system. The phase space *Γ* is a space of dimension 2M, where *M* can be on the order of $10^{23}$ for macroscopic systems. Boltzmann’s key insight was that a macroscopic observer cannot distinguish between certain microscopic states. Such an observer describes the state of the system using macroscopic variables that are unaffected by specific details of the microstate. As a result, a macroscopic state A is compatible with a set of microstates Γ(A)⊂Γ. Boltzmann then proposed the ergodic hypothesis, which asserts that a large system with Hamiltonian H(x) explores the energy shell $\gamma_{E}\equiv \{x\;:\;H(x)=E\}\subset \Gamma$—the set of microstates with energy *E*—uniformly. Consequently, the probability of finding the system in a macrostate A is proportional to the volume^a^  ω(A) of the set $\Gamma (A)\cap \gamma_{E}$ of compatible microstates in the energy shell.

Moreover, for typical macrostates, the values of ω(A) vary greatly from one macrostate to another. Hence, the system will almost certainly evolve towards the most likely macrostate $A^{\mathrm{eq}}$, which is the one that maximizes ω(A). In fact, the set $\Gamma (A^{\mathrm{eq}})$ of all the microstates compatible with macrostate $A^{\mathrm{eq}}$ occupies almost all the energy shell and, consequently, it will be observed with probability close to one.

Based on Boltzmann’s ideas, Planck introduced the celebrated expression for the entropy of a macrostate, S(A)=klnω(A), where $k≃1.38\times 10^{-23}$ J/K is the Boltzmann constant. The logarithm ensures the additivity of entropy and, since it is an increasing function, the ergodic hypothesis guarantees that the system tends toward the macrostate with the highest entropy.

## Observable-dependent entropy

One can avoid the arbitrariness in the definition of macrostates by reformulating Boltzmann’s argument in terms of particular observables. This particularization is implicit in Einstein’s studies of fluctuations (4) and in other presentations of statistical mechanics (20).

We introduce an entropy function associated with an arbitrary observable A(x). First, consider the volume of the set of microstates within the energy shell that are compatible with a given value *a* of the observable:


(1) $$\omega_{A}(a;E)\equiv \int_{\Gamma }\;dx\;\delta (E-H(x))\delta (a-A(x)).$$

Then, we define the entropy function associated to observable *A* as


(2) $$S_{A}(a;E)\equiv k\ln\;\omega_{A}(a;E).$$

If the system is ergodic, i.e. if it uniformly explores all microstates with energy *E*, then the probability density of the outcome of a measurement of observable *A* is^b^


(3) $$\rho_{A}(a)=\frac{\omega_{A}(a;E)}{\omega (E)}\propto e^{S_{A}(a;E)/k},$$

where


(4) $$\omega (E)\equiv \int_{\Gamma }dx\;\delta (E-H(x)).$$

More precisely, the ergodic property implies that, if we let the system evolve for a long time *τ*, the probability of observing at a given time a value of *A* within the interval [a,a+da] is $\rho_{A}(a)da$.

The above definitions and Eq. (3) can be extended to several observables, and the latter is known as Einstein’s formula for fluctuations (4, 20). Notice that the entropy $S_{A}(a;E)$ defined by (1) and (2) is entirely objective, i.e. does not depend on any potential observer and can be applied to any observable A(x), either microscopic, like the velocity of a single molecule, or macroscopic.

Under the ergodic hypothesis, the probability distribution $\rho_{A}(a)$ is also objective. If we let the system evolve and register the value of the observable A(x(t)) during an interval of time [0,τ], then $\rho_{A}(a)$ is the empirical probability density function (pdf) of these values in the limit τ→∞. Nevertheless, this limit indicates that the distribution $\rho_{A}(a)$ describes the fluctuations of the system *only at equilibrium*, since the pdf when τ→∞ does not depend on data within a finite interval, such as the transient regime before the system reaches equilibrium. Hence, the ergodic hypothesis is useless for describing or assessing any irreversible relaxation to equilibrium. Nonetheless, it is indeed useful for explaining irreversibility.

## Objectivity of irreversibility

Irreversibility arises when the initial value $a_{0}=A(x(0))$ has a low entropy, i.e. when $a_{0}$ is very unlikely, according to (3). Then the observable *A* evolves toward higher-entropy values. We now introduce a first criterion for irreversibility, which we will later qualify, but it captures several essential aspects of irreversible behavior.

We say that observable *A* exhibits irreversible behavior if


(5) $$S_{A,\max}-S_{A}(a_{0};E)\gg k,$$

where $S_{A,\max}=\max_{a}S_{A}(a;E)$. More precisely, the irreversible relaxation will drive *A* toward values *a* such that $S_{A,\max}-S_{A}(a;E)≲k$. If we denote by $a^{\mathrm{eq}}\equiv \mathrm{argmax}\;S_{A}(a;E)$ the most likely value, then *A* irreversibly evolves toward values close to $a^{\mathrm{eq}}$. Here, “irreversible” means that the reverse process, i.e. the observation of a departure from values of *A* close to $a^{\mathrm{eq}}$ to values with low entropy, is very unlikely (21).

The criterion (5) for observing an irreversible behavior *in a given observable A* is not precise. This is expected, since irreversibility is always masked by fluctuations, which are more noticeable for microscopic observables. Nevertheless, the absence of a sharp boundary between irreversible and reversible behavior should not be mistaken for subjectivity or arbitrariness. Our criterion (5) provides an objective (albeit coarse) distinction between the two behaviors, which crucially does not depend on any external observer.

When the observable is macroscopic, the entropy function $S_{A}(a;E)$ is extensive and $\rho_{A}(a)$ approaches a delta distribution centered at the most likely value $a^{\mathrm{eq}}$, $S_{A,\max}=S_{A}(a^{\mathrm{eq}};E)$, which can be identified with the equilibrium value. In this case, the theory predicts a quasideterministic, irreversible relaxation toward $a^{\mathrm{eq}}$, independent of the initial condition.

By contrast, the irreversibility criterion introduced above applies to any observable, whether microscopic or macroscopic, as we illustrate in the next section, thereby clarifying the crossover between these two regimes.

## Surprises: irreversibility at the microscale

The criterion for irreversibility (5) yields some interesting and somewhat unexpected results when applied to microscopic observables.

Let us calculate the entropy (2) of some microscopic observables in a classical, monoatomic ideal gas with *N* molecules. Consider the observable $X_{1}$, the *x*-coordinate of a given molecule. It is relatively easy to show that the entropy function $S_{X_{1}}(x;E)$ is constant. Therefore, the criterion (5) can never be achieved by the entropy associated with $X_{1}$. Hence, the position of a single molecule in a gas cannot exhibit an irreversible behavior.

Consider, on the other hand, the kinetic energy of the molecule, $E_{1}=|p_{1}{|}^{2}/(2m)$. It is a simple exercise to calculate the entropy $S_{E_{!}}(\epsilon ;E)$ and the corresponding probability distribution using (3) and the definitions (1) and (2). The result is


(6) $$\begin{array}{rl} \rho_{E_{1}}(\epsilon ) & =N\;\epsilon^{1/2}{\left(E-\epsilon \right)}^{(3N-5)/2}≃N\sqrt{\epsilon }\;\exp\;\left(-\frac{3N\epsilon }{2E}\right), \end{array}$$

where N is a constant. One recognizes the probability distribution for the energy of a single particle that derives from the Maxwell velocity distribution with temperature T=2E/(3Nk). Now, suppose that a given molecule acquires a kinetic energy of, say, 1,000kT. This can occur, for instance, due to a collision with a cosmic high-energy particle. After the collision, the molecule will slow down from an initial energy $\epsilon_{0}=1,000\;kT$ to kinetic energies near *kT*. This is an irreversible relaxation at the microscale. On the other hand, if the gas is at equilibrium (homogeneous density and velocities distributed according to the Maxwell distribution), the macroscopic properties of the gas do not change. In this example, the microscopic world exhibits an instance of irreversible behavior, whereas the gas behaves reversibly at the macroscale. Notice, however, that the molecule is immersed in a system with many degrees of freedom, which are necessary to observe the irreversible relaxation.

We can indeed call irreversible this relaxation of the molecule’s kinetic energy in precisely the same way that we refer to breaking a glass into many tiny pieces as irreversible. The time-reversed behavior—the spontaneous recollection of the pieces to form a glass—is theoretically possible, according to Newtonian mechanics, but the probability of such an event is extremely small. Similarly, the molecule in our example could, in principle, acquire a kinetic energy of 1,000kT from collisions with other gas molecules, but the probability of such a fluctuation is of the order of $e^{-1,000}$.

Notice that there is no conceptual difference between the irreversibility exhibited by macroscopic and microscopic quantities, except for the magnitude of their respective fluctuations.

## Objections: the choice of observables

**Figure 1:**
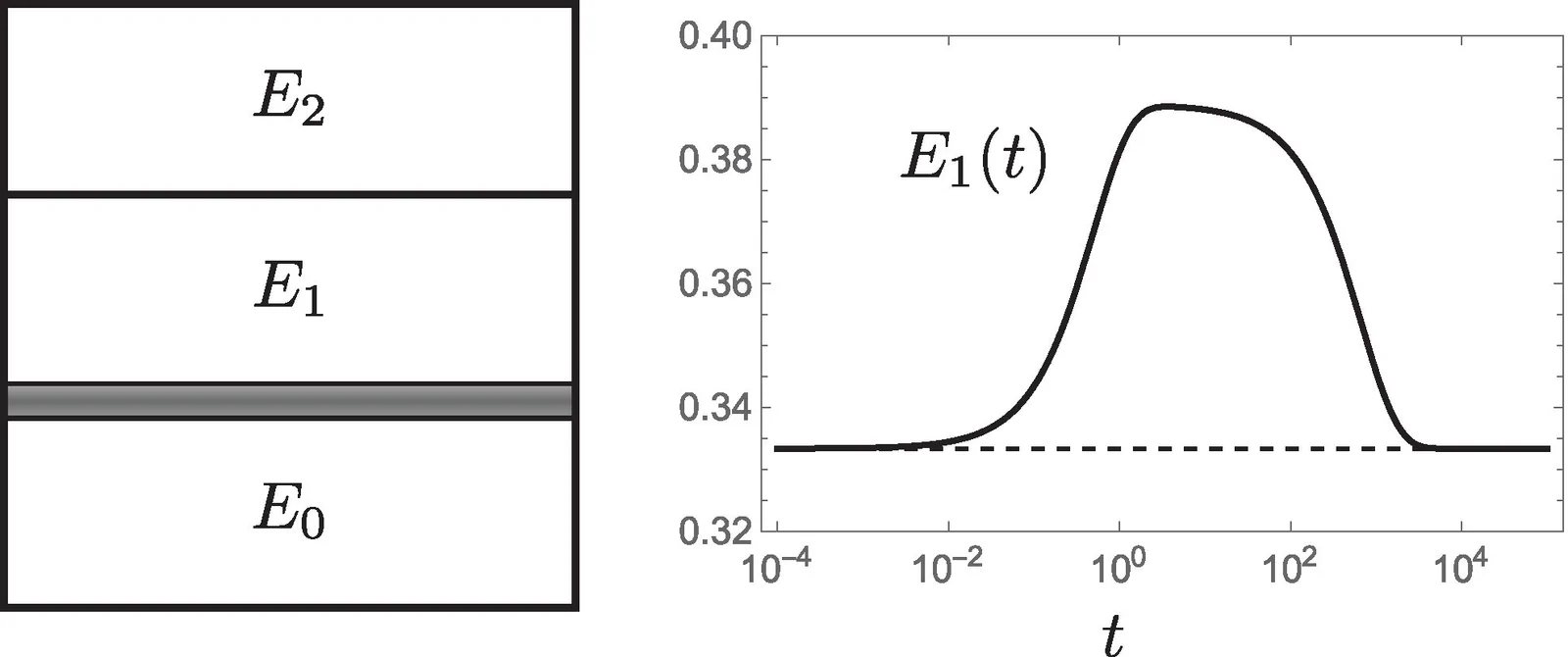
Three identical gases exchange energy, but the thermal conductivity of the wall separating gases 0 and 1 is much smaller than the one separating gas 1 and gas 2. The rightmost plot shows the energy of gas 1, $E_{1}(t)$, for the initial condition $E_{0}(0)=4/9$, $E_{1}=1/3$, and $E_{2}=2/9$, in units where the total energy is E=1.

As already mentioned, the criterion for irreversibility (5) is insufficient in some situations and even misleading. In particular, the choice of observables is crucial for its validity. Consider the following situation: three identical gases, labeled 0, 1, and 2, exchange energy through walls, as sketched in Figure 1. The conductivity of the wall separating gases 0 and 1 is much smaller than the one separating gases 1 and 2. As a consequence, gases 1 and 2 equilibrate in a much shorter time scale than gases 1 and 0. Suppose now that the initial energies are $E_{0}=2/9$, $E_{1}=1/3$, and $E_{2}=4/9$, in units such that the total energy is E=1. The energy of gas 1, $E_{1}(t)$, departs from its most likely value $E_{1}^{\mathrm{eq}}=1/3$, because it thermalizes with gas 2 at $E_{1}≃(E_{1}+E_{2})/2=7/18≃0.39$ and then returns to the initial value at longer times, as shown in the rightmost plot of Fig. 1.

The example shows that $S_{E_{1}}(\epsilon ;E)$ and criterion (5) are insufficient to predict the irreversible behavior of $E_{1}$. It is necessary to consider two observables, $E_{1}$ and $E_{2}$, for instance, and realize that $S_{E_{1},E_{2}}(\epsilon_{1},\epsilon_{2};E)$ is highly peaked around the point $\epsilon_{1}=\epsilon_{2}=E/3$. The effect of hidden degrees of freedom on the relaxation dynamics of meso- and macroscopic systems is known as the Kovacs effect, which has been extensively studied in recent years both theoretically (22) and experimentally (23). Notice that this effect does not compromise the objectivity of the irreversible behavior, but only the validity of criterion (5) to predict it.

## Objectivity of equilibrium and entropy

Equilibrium is identified with the total absence of irreversibility. Accordingly, one might attempt to define a microstate $x_{0}$ as an equilibrium state if it does not exhibit irreversibility in any observable A(x). However, this definition immediately fails. Consider, for instance, the observable


(7) $$A(x)=d(x,x_{0}),$$

where d(x,y) is a distance defined on the phase space *Γ* (e.g. the Euclidean distance). If the system starts at $x_{0}$, this observable will typically evolve from $A(x_{0})=0$ to some value close to $a^{\mathrm{eq}}$, the value that maximizes the entropy $S_{A}(a;E)$. Thus, the arbitrary microstate $x_{0}$ is “out of equilibrium” with respect to some admissible observable.

One possible way to overcome this difficulty and define equilibrium microstates is to restrict the set of observables that are considered legitimate for describing the system. This strategy underlies most attempts to define equilibrium (19, 24). Yet any restrictive choice of observables seems to introduce a degree of subjectivity or arbitrariness.

Importantly, this lack of objectivity in the definition of equilibrium states does not preclude the fact that a given observable may reach an equilibrium value in an objective, observer-independent manner. For example, the spectrum of the radiation contained in a cavity whose walls are kept at temperature *T* converges to Planck’s law. This relaxation is objective, even if there is no observer-independent way to define an equilibrium state of the electromagnetic field inside the cavity.

An analogous situation arises with entropy. The *entropy of a system* is the entropy associated with a chosen set of observables, and the selection of these observables is arbitrary. Entropy is not a property of the system alone, but of a particular description of the system.

The difference between objective and subjective concepts is subtle (5). The entropy associated with a given observable is an objective quantity, much like the velocity of a particle or the total kinetic energy of a gas. *Given* an observable *A*, its entropy function $S_{A}(a,E)$ is unambiguously defined by Eq. (2). The irreversible behavior of a given observable is also objective, as well as the fact that the associated entropy increases. On the other hand, the *entropy of a system* cannot be defined unless we specify which observables determine the state of the system. The element of subjectivity or arbitrariness is precisely the choice of observables.

As already mentioned, the only way to remove this arbitrariness would be to provide an objective criterion for selecting such observables. One could restrict the choice to macroscopic observables, those with extensive associated entropy, as Lebowitz suggests (3). However, this criterion is overly permissive. For example, in a classical gas with *N* molecules, the center of mass of any subset of N/2 molecules is a macroscopic observable. One can choose the center of mass of the subset of molecules located in the left half of the container, which will exhibit irreversible relaxation even if the gas is in equilibrium. A second condition could be imposed, namely, invariance under permutations of particles. This criterion is used by Pitowsky (24) to propose an objective definition of equilibrium. However, it has not yet been proven that this would completely resolve the problem.

Moreover, it is not clear why microscopic observables should be excluded from the description of a system’s state. In stochastic thermodynamics, for instance, it is common to take the microstate of a system in contact with a thermal bath and the bath’s energy (or equivalently, its temperature) as relevant observables (25). In that case, the arbitrariness lies in the partition of a global system into a system of interest and a reservoir or environment.

In any case, to my knowledge, there is no clear objective criterion for selecting the observables that determine a system’s state. I think it is better to admit this fact and conclude that the entropy of a system and the notion of equilibrium depend on the chosen description of the system, which can change according to our interests or technical capabilities.

These considerations raise a provocative question: are the definitions of equilibrium state and system entropy really useful or necessary? Entropy and equilibrium for *given observables* are objective and appear to offer the same explanatory power as entropy and equilibrium for systems, which depend on the choice of observables. The example of thermal radiation illustrates this point clearly: Planck’s law is objective, whereas the field’s equilibrium state is not.

## Objectivity of temperature

If an isolated system is composed of two subsystems, *A* and *B*, whose interaction energy can be neglected, then the entropy associated with the energy of system *A* can be written as


(8) $$S_{E_{A}}(\epsilon ;E)=S_{A}(\epsilon )+S_{B}(E-\epsilon ),$$

where


(9) $$S_{i}(\epsilon )\equiv k\ln\;\int_{\Gamma_{i}}dx_{i}\delta (E-H_{i}(x_{i}))$$

is the microcanonical entropy of subsystem i=A,B with energy ϵ. In this expression, $x_{i}$ represent a microstate of system i=A,B, and $\Gamma_{i}$ and $H_{i}(x_{i})$ its phase space and Hamiltonian, respectively. Then, $\epsilon^{\mathrm{eq}}$, the most likely value of $E_{A}$ verifies:


(10) $${\frac{\partial S_{A}(\epsilon )}{\partial \epsilon }|}_{\epsilon =\epsilon^{\mathrm{eq}}}={\frac{\partial S_{B}(\epsilon )}{\partial \epsilon }|}_{\epsilon =E-\epsilon^{\mathrm{eq}}}.$$

With the standard definition of temperature:


(11) $$T_{i}(\epsilon )\equiv {\left[\frac{\partial S_{i}(\epsilon )}{\partial \epsilon }\right]}^{-1}.$$

Equation (10) reads $T_{A}(\epsilon^{\mathrm{eq}})=T_{B}(E-\epsilon^{\mathrm{eq}})$.

The temperature defined in (11) is an objective quantity that determines the direction of the energy flow between the two systems. The only assumption made here is the additivity of the entropy associated with the energy of system *A*, Eq. (8).

It is well known that the derivative of entropy with respect to energy can yield different values depending on which other quantities are held fixed (26). Since we are analyzing the properties of observable-dependent entropy, one could ask for the physical relevance of the derivative in (11) when using the entropy associated with the energy $E_{A}$ and other observables of system *A*. In the Supplementary material, we present an example illustrating this possibility. The conclusion, as expected, is that the argument above is valid as long as one can distinguish in an isolated system two parts, *A* and *B*, with well-defined energies $E_{A}$ and $E_{B}$, and the entropy associated with $E_{A}$ or $E_{B}$ is additive, as in Eq. (8). Under these conditions, we can ensure that the temperature defined in (11) indicates the direction of the energy flow between the two systems and coincides with the standard thermodynamic temperature.

## The origin of low-entropy states and the past hypothesis

Returning to the problem of irreversibility, the remaining question is how an observable *A* can take a value $a_{0}$ with such a low entropy that its spontaneous occurrence is practically impossible.

The conventional answer is that the universe began in a state of very low entropy—the so-called *past hypothesis* (2)—and that this low entropy somehow propagated across galaxies, stars, and planets, thereby creating low-entropy environments and, eventually, low entropy systems such as experimental physicists, who can prepare systems in initial states with arbitrarily low entropy. The past hypothesis has been criticized in several ways (2, 15). Perhaps its main difficulty lies in defining or assessing the entropy of the entire universe.

Here, I suggest a specific mechanism for the origin of low-entropy states based on symmetry breaking. It replaces the past hypothesis with a milder—and empirically well-supported—assumption: that cosmic expansion produces an environment with decreasing temperature, namely the cosmic microwave background radiation. It also differs from the picture of *branch systems* introduced by Reichenbach: systems that reach low-entropy states through interaction with ordered environments (6, 15).

**Figure 2:**
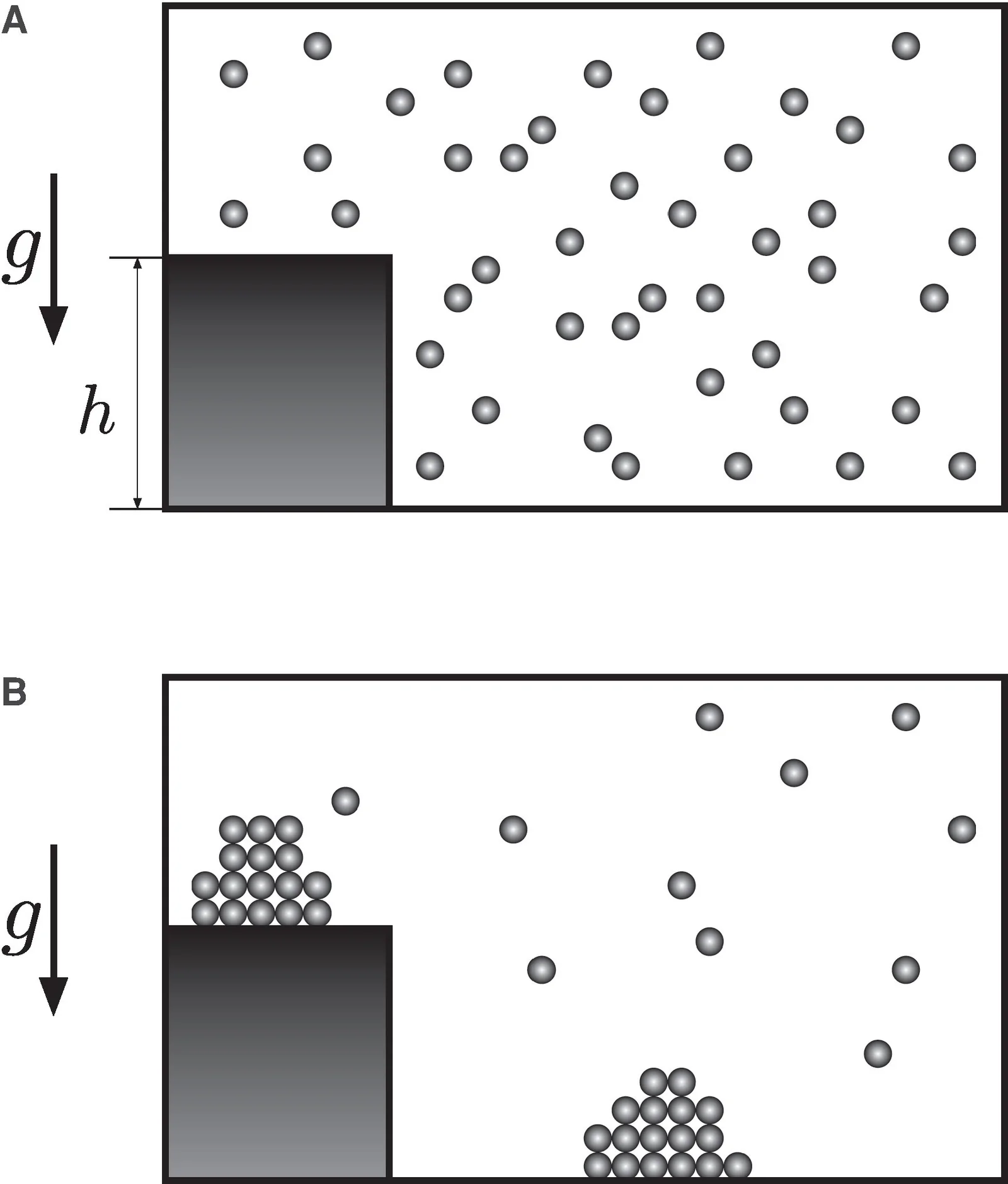
The origin of a low-entropy state in a system consisting of a gas in a room below the condensation temperature. A) In the room, there is a shelf at a height *h* sketched as a dark rectangle. B) Condensation can occur on the shelf or on the floor of the room; i.e. a drop can form at a height *z*, with z=0 or z=h. The probability of these two possibilities is of the same order. In contrast, the entropy of the former compared to the latter is ΔS∼mgh/T, which means that the ratio of the volumes of the regions compatible with each value of *z* is $e^{\Delta S/k}∼e^{mgh/(kT)}$. This is of the order of $\exp\;(10^{18})$ for a drop of 1 gram at 300 K and with h=50 cm. When the drop forms on the shelf, a fluctuation of the order $\Delta S∼k\times 10^{18}$ has occurred in the system.

I will introduce this mechanism with two simplified examples. The first one is a gas in a box with a shelf at a height *h* (see Fig. 2). The gas cools below its condensation temperature. When this happens, droplets may form on the floor or on the shelf with comparable probability. However, the entropy $S_{Z}(z;E)$ associated with the observable *Z*, defined as the height of the center of mass of the droplet, is vastly larger for a droplet forming on the floor than for one forming on the shelf. A simple estimate gives the difference of entropies $\Delta S\equiv S_{Z}(0;E)-S_{Z}(h;E)∼mgh/T$, where *T* is the temperature of the gas, defined as kT=2(E−mgz)/3, *m* the mass of the droplet, and *g* the gravitational acceleration. For a droplet of 1 g, h= 1 m and T=300 K, this yields $\Delta S≃k\times 10^{18}$. This means that the region of phase space compatible with the droplet forming on the floor is roughly $\exp\;(10^{18})$ times larger than the region compatible with a droplet forming on the shelf.

This comparatively tiny region of phase space is nonetheless occupied with a non-negligible probability because condensation is a symmetry-breaking transition: the droplet can nucleate anywhere within the box, and the transition effectively partitions the phase space into regions that are not dynamically connected. As a result, ergodicity breaks down and each region is reached with a probability determined by the condensation dynamics rather than by its phase-space volume (27).

**Figure 3:**
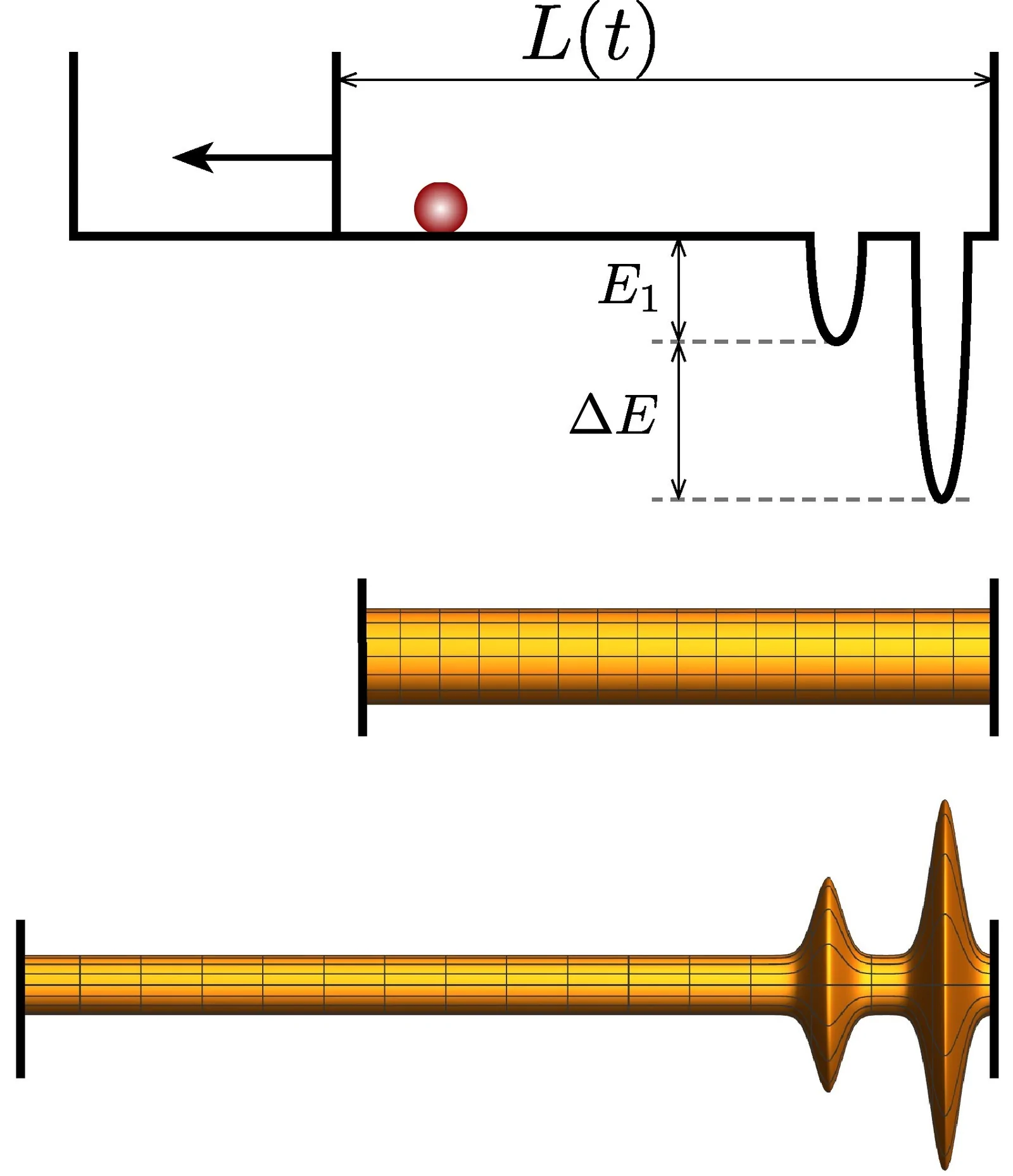
A specific example of the creation of a low-entropy metastable state. A Brownian particle in a box of length L(t) is subjected to a potential with two wells of depth $E_{1}$ and $E_{2}=E_{1}+\Delta E$, respectively (top panel). The box contains a gas that cools down due to the adiabatic expansion caused by quasistatically moving the leftmost well to the left. The energy shell deforms as sketched in the two bottom panels.

The second example is a 1D Brownian particle in a potential with two wells with different depths, $E_{1}$ and $E_{2}=E_{1}+\Delta E$, as shown in Fig. 3. The particle is immersed in a gas that undergoes an adiabatic expansion, with the length of the container increasing as L(t). The expansion cools down the gas. Let us assume that the initial temperature of the gas, $T_{0}$, is much higher than $E_{2}/k$, whereas the final temperature, $T_{f}$, is much lower than $E_{1}/k$. In this case, during the expansion, the particle is trapped in the shallower well with probability close to 1, even though the entropy associated with its position is much higher for the deepest well. If $x_{1}$ and $x_{2}$ are the locations of the shallow and deep wells, respectively, then the entropy $S_{X}(x;E)$ associated with the position *X* of the particle obeys, approximately


(12) $$\Delta S\equiv S_{X}(x_{2};E)-S_{X}(x_{1};E)=\frac{\Delta E}{T}.$$

If ΔE is, say, of the order of millijoules and $T_{f}$ of the order of hundreds of Kelvin, then $\Delta S/k∼10^{18}$. As in the previous example, the system has spontaneously reached a low-entropy state. The lower pane of Fig. 3 is a sketch of the behavior of the region in phase space compatible with *x* and *E*. This region is a hyper-sphere of dimension $2N_{\mathrm{gas}}$, where $N_{\mathrm{gas}}$ is the number of molecules in the gas, and of radius proportional to $\sqrt{E-V(x)}$, V(x) being the potential energy of the Brownian particle. Initially, the entropy $S_{X}(x;E)$ is approximately constant. When the gas cools down, the radius of the hyper-sphere decreases, except for $x≃x_{1},x_{2}$. The volume of the region compatible with $x=x_{2}$ is vastly larger than the one compatible with $x=x_{1}$, by a factor of order $\exp\;(10^{18})$. Nevertheless, the system gets trapped in the region compatible with $x=x_{1}$.

The previous examples show that ergodicity breaking can occur due to phase transitions or potential barriers exceeding *kT*. The first ones, especially in the case of breaking of continuous symmetries, are responsible for the appearance of macroscopic and slow degrees of freedom, like the position of the droplet (the position of any meso- or macroscopic object is the result of breaking translational symmetry). The second ones are responsible for the stability of nuclei, molecules, and complex biomolecules (27).

The mechanism for generating low-entropy states that I have introduced here depends on the external driving that lowers the system temperature, thereby allowing symmetry or ergodicity breaking. The mechanism is different from the spontaneous occurrence of a giant fluctuation. The latter is the time-reversal of a relaxation (6). On the other hand, here we have a true time-reversal asymmetry: the low-entropic state is generated by symmetry breaking, and the eventual subsequent relaxation to a state of higher entropy (for instance, the transition from the shallow to the deep well in the example of Figure 3) is different from the time-reversal of the symmetry breaking. The time-reversal asymmetry is induced by a driving that can be quasistatic, i.e. it is not the result of a nonequilibrium driving producing entropy. Instead, it is the result of a reconfiguration of the phase space that *dynamically* favors regions that are tiny compared with other regions.

The induced irreversibility is analogous to the one observed in a hysteretic cycle. When the magnetic field is driven from a high positive value to a moderate negative value, the system remains in a low-entropy, positive-magnetization state that cannot be reached by spontaneous fluctuations. This metastable state relaxes when the field is sufficiently intense, and the relaxation is highly dissipative and irreversible.

The example depicted in Figure 3 suggests that an adiabatic expansion, such as the one experienced by the cosmic background radiation, could be sufficient to generate low-entropy states. In this view, the controversial “past hypothesis” (2)—namely, the assumption that the universe began in a state of extremely low entropy—may be replaced by the simpler idea of an expanding universe that creates a reservoir of decreasing temperature (the background radiation). This reservoir, in turn, induces symmetry-breaking transitions that generate macroscopic and slow degrees of freedom in low-entropy states. It should be emphasized, however, that other mechanisms may also contribute to the generation of low-entropy states. Reichenbach’s branch systems (6), for instance, may play an important role in the “propagation” of low entropy.

Finally, it is worth noting that driving is usually interpreted as the action of an external agent, but it can also be implemented by a degree of freedom with a large mass and nonzero initial velocity (28). With this interpretation, driven systems become autonomous Hamiltonian systems. This is in fact, the cause of most “natural” driving forces: for instance, the periodic driving of the intensity of sunlight reaching a point at the surface of the earth is due to the rotation of the earth, i.e. to a degree of freedom (the rotation angle) with large mass (in this case, large moment of inertia) and a nonzero initial velocity.

## Traces

Another fundamental problem concerning the arrow of time and its objectivity is the relation between records, footprints, or traces, and the increase of thermodynamic entropy (6–9, 29). This issue is closely related (if not identical) to the so-called psychological arrow of time, namely, that we remember the past and can influence the future. Many researchers claim that the psychological arrow of time derives from the thermodynamic arrow of time, i.e. that the creation of a trace of an event necessarily entails an increase in entropy (7). However, alternative viewpoints exist. Hemmo and Shenker maintain that the psychological arrow of time is fundamental, with the thermodynamic time asymmetry emerging from it (8). Gołosz, by contrast, argues that neither arrow explains the other, and that both the psychological and thermodynamic arrows originate from a deeper, more fundamental arrow of time (9).

In this section, I show that traces are indeed accompanied by an increase in entropy in isothermal processes, provided one considers not only the physical creation of the trace but also the generation of its informational content and the eventual erasure of that information (30). The approach is based on the observation that symmetry-breaking transitions generate macroscopic randomness or macroscopic information (27, 30).

Suppose we record a movie of a shoe leaving a footprint in the snow, which is later erased by the wind. If the movie is played backward, two distinct quasi-impossible phenomena appear. First, the spontaneous formation of the footprint, a highly structured pattern, in the snow. Second, the fact that this spontaneously formed pattern matches *exactly* the sole of the shoe that would later step on it. The two phenomena are astronomically improbable, indicating that they both contribute to the irreversibility of the whole process. However, this is not completely true: we will see that the first one, the spontaneous formation of the pattern, does not necessarily imply any irreversibility.

To make this intuition more transparent, consider a simplified analog of the footprint process. Footprinting is essentially the transfer—or copying—of information from one system (the sole) to another (the snow). A similar process can be envisioned in a ferromagnetic material. Suppose that a piece of ferromagnetic material is cooled below its Curie temperature and acquires a nonzero magnetization. For simplicity, let this magnetization be a scalar taking only two possible values, positive or negative (30). We then magnetize a second piece of material using the magnetic field produced by the first, so that both end up with parallel magnetizations. Finally, the pieces are separated and heated above their Curie temperature, causing both magnetizations to vanish.

The backward movie of this process would show two bits of information, namely, the magnetization directions, appearing spontaneously and coinciding. The probability of such a coincidence is 1/2, which is still too high for an observer to determine the temporal direction of the process reliably.

Now consider the same process involving a material with *N* independent magnetic domains. After cooling below the Curie point, the material acquires *N* random bits of magnetization. These bits are then copied to another *N*-domain material, and the two pieces are moved far apart and heated up. In the backward movie, we would observe both materials being cooled below their Curie temperature and spontaneously generating *N* random bits each, a perfectly ordinary event.

What is quasi-impossible in the backward process is that the two independently generated strings of *N* bits coincide exactly. This is the equivalent of the coincidence of the spontaneous footprint and the pattern of the sole. In the case of the *N* bits, the probability of this coincidence is $2^{-N}$. Observe that the irreversibility relies here on the coincidence of the two strings of bits, but not on the creation of the random string.

One can calculate the entropy change in an analogous process, using a simple model of a ferromagnet (3). For that purpose, it is more convenient to model the copying of information stored in the domains’ magnetization as an isothermal process. Instead of changing the temperature to induce the symmetry breaking, one can increase the coupling between spins above its critical value (30), keeping the temperature *T* constant. The process is a concatenation of isothermal processes in which an external agent changes the coupling $J_{A}$ between spins of system *A*, the coupling $J_{B}$ between spins of system *B*, and the coupling $J_{AB}$ between spins in *A* and *B*. Initially, $J_{A}=J_{B}=J_{AB}=0$ and the magnetization in the two systems is zero. The process consists of the following steps: (i) the external agent increases $J_{A}$ above its critical value and system *A* acquires a random magnetization, (ii) the agent increases $J_{B}$ and $J_{AB}$ and system *B* acquires the same magnetization as *A*, (iii) the intersystem coupling $J_{AB}$ is decreased back to zero, (iv) the intrasystem couplings $J_{A}$ and $J_{B}$ are decreased back to zero. Information is created in (i), copied in (ii), and erased in (iv). Notice that step (iv) is different from the information erasure that obeys Landauer’s principle. In particular, it does not imply any extra heat dissipation (10).

Using stochastic thermodynamics (31, 32) or an explicit calculation with globally coupled Ising models, one can calculate the minimal work that the external agent must perform to copy one bit (30). This minimal work turns out to be kTln2 and equals the dissipated heat. Since the process is a cycle, the minimal entropy production is $S_{\mathrm{prod}}=k\ln\;2$. Here, the entropy production is given by the standard Clausius equation and is equal to the change in the entropy $S_{E_{A},E_{B}}(\epsilon_{A},\epsilon_{B};E)$ associated with the energies of the two subsystems *A* and *B*.

For *N* bits, the entropy production is $S_{\mathrm{prod}}=Nk\ln\;2$. If the process is run backward, the probability that the *N* bits coincide is precisely $\exp\;(-S_{\mathrm{prod}}/k)=2^{-N}$. This equality reflects a profound relationship between irreversibility and entropy production, which has been explored in the context of stochastic thermodynamics (27, 31, 32). For systems in contact with reservoirs, one can prove that entropy production is proportional to a quantitative measure of irreversibility, namely the Kullback–Leibler divergence between the probability distributions of the probabilistic states in a process and in its time reversal (31–33). This result shows that any irreversibility, i.e. any possibility of distinguishing, even in a probabilistic manner, between a process and its time reversal, is accompanied by an increment of entropy (27, 31, 32).

Interestingly, if one swaps steps (c) and (d) in the process, i.e. if the information is erased when the two systems are still in contact, the entropy production vanishes (30). Irreversibility also disappears: that backward process consists now of the increase of $J_{A}$ and $J_{B}$ with $J_{AB}$ positive. This means that the magnetization of the two systems is the same. This point is crucial to understanding the irreversibility of copying information. One must consider the entire process: information creation, copying, and erasure *after decoupling the two systems*. This process exhibits an arrow of time that coincides with the increase of observable-dependent entropy.

## Objectivity of the second law: ergotropy

Finally, in this section, I discuss the objectivity of the second law of thermodynamics. One popular statement of the second law asserts that the entropy of an isolated system can only increase over time. Accordingly, Boltzmann’s argument for the increase in entropy is often considered a rationale for the second law.

However, the definition of entropy out of equilibrium remains controversial (34, 35). For this reason, most thermodynamics textbooks formulate the second law, directly or indirectly, in terms of the impossibility of certain processes (see Refs. (21, 36) for a detailed discussion of the different formulations of the second law and their relation to irreversibility). For instance, the Kelvin–Planck statement asserts that it is impossible to construct an engine that operates in a complete cycle and produces no effect other than raising a weight and cooling a heat reservoir.

The objectivity of this formulation of the second law was questioned by Maxwell through his celebrated demon (10, 11). Maxwell’s demon shows that, if an external agent possesses or acquires information about thermal fluctuations in a system in contact with a thermal bath, it could extract energy from the bath in a cycle. The demon therefore suggests that the validity of the second law depends on the capabilities of the agent manipulating the system. In this view, the second law would no longer be an objective physical principle but rather a statement about the inability of certain observers to implement particular processes.

The strategy for eliminating this apparent subjectivity is to consider the demon’s physical nature. Then one finds that the information processing required for the demon to accomplish its task necessarily entails energetic costs that restore the validity of the second law, regardless of the demon’s capabilities. The second law thus recovers its status as an objective physical principle (10, 11).

However, the energetic costs associated with information processing, such as those described by Landauer’s principle (10), have been investigated primarily in scenarios where both the demon and the system are in contact with thermal baths or reservoirs, under certain assumptions such as weak coupling and Markovianity or initial conditions where the system and the reservoirs are uncorrelated (25). A more general approach applicable to arbitrary Hamiltonian systems is desirable.

The enlarged system comprising the system and the demon must be externally driven, since the different operations of the machine—measurement, feedback, erasure of the measurement outcome, or resetting the demon’s memory—require protocols that modify parameters of the global system, such as the coupling between the demon and the system. From this perspective, the problem posed by Maxwell’s demon is shifted to the question of whether an external agent can extract work from a system without performing measurements, that is, by implementing nonfeedback protocols (11).^c^

This question can be stated in purely mechanical terms, considering an external agent that manipulates the Hamiltonian of a system whose initial state is stochastic. To characterize this possibility, Allahverdyan, Balian, and Nieuwenhuizen introduced in Ref. (14) the concept of *ergotropy*.

The ergotropy of a probabilistic state ρ(x) with respect to a Hamiltonian H(x) is defined as


(13) $$W(\rho )=\max_{U}\int_{\Gamma }dx\;H(x)\left[\rho (x)-\rho (U^{-1}(x))\right],$$

where the maximization is done over all possible canonical maps U on *Γ*, i.e. over any arbitrary Hamiltonian evolution that drives the system from a microstate *x* to U(x). Then, W(ρ) is the maximum average work that an external agent can extract from the system in a cycle.

Decades earlier, Gorecki and Pusz (13) introduced the notion of passive states in classical mechanics, referring to probability distributions ρ(x) for which W(ρ)=0, i.e. states from which it is impossible to extract energy. In other words, passive states are those that comply with the Kelvin–Planck statement of the second law. They also proved that a state ρ(x) is passive if and only if $\rho (x)=f(H(x;\lambda_{0}))$, where f(⋅) is a nonincreasing function. In particular, the canonical ensemble or Gibbs state is passive, while the microcanonical state is not. In principle, energy can be extracted from a system in the microcanonical state. There are examples of this extraction (40, 41), but all of them are systems with one degree of freedom, and the extension to several degrees of freedom is under question (40).

If the microcanonical state were passive, then we would have a plausible rationale for the second law, combining the impossibility of extracting energy in a cyclic protocol and Boltzmann’s ergodicity for equilibration. We can state that, if a system is isolated for a sufficiently long time, its microscopic state is a random variable uniformly distributed within the energy shell. Then, it would be impossible to extract energy, on average, via a cyclic manipulation.

Even though the microcanonical state is not passive, there are strong reasons to believe that the energy that can be extracted from this state is minute. For a state ρ(x) that is uniform over a set A∈Γ, the minimum in (13) is reached by a canonical transformation that maps the set *A* into the set $\Gamma_{E}\equiv \{x\in \Gamma \;:\;H(x)\leq E\}$ enclosed by an energy shell.

We can apply this result to a microcanonical ensemble for which the energy is known with some uncertainty ΔE:


(14) $$\rho (x)=\frac{\Theta (E_{0}-H(x))-\Theta (E_{0}-\Delta E-H(x))}{\phi (E_{0})-\phi (E_{0}-\Delta E)},$$

where Θ(x) is the Heaviside step function and


(15) $$\phi (E)\equiv \int_{\Gamma }dx\;\Theta (E-H(x))$$

is the phase-space volume of $\Gamma_{E}$.

If we can map all the points in the energy layer $\left\{x\;:\;H(x)\in [E_{0}-\Delta E,E_{0}]\right\}$ onto the set $\Gamma_{E_{1}}=\{x\;:\;H(x)\leq E_{1}\}$, we get the final passive state


(16) $$\rho_{f}(x)=\frac{\Theta (E_{1}-H(x))}{\phi (E_{1})}.$$

Liouville’s theorem implies that the final energy $E_{1}$ satisfies


(17) $$\phi (E_{1})=\phi (E_{0})-\phi (E_{0}-\Delta E).$$

For the microcanonical state of an ideal gas with *N* particles, $\phi (E)\propto E^{M}$, with M=3N/2. Hence, the maximum fraction of energy that we can extract from state (14) is:


(18) $$\frac{E_{0}-E_{1}}{E_{0}}≃1-{\left[1-e^{-M\Delta E/E_{0}}\right]}^{1/M}.$$

To extract a significant fraction of energy, it would be necessary that $M\Delta E/E_{0}$ is small. Then, we can approximate the expression between brackets as $(M\Delta E/E_{0}{)}^{1/M}$. This is significantly <1 only if $M\Delta E/E_{0}≲e^{-M}$ which means a ridiculous accuracy in the original energy even for a mesoscopic system with N=20 particles and M=30.

In conclusion, the ergotropy of the microcanonical ensemble is negligible unless the protocol is designed for a specific value of the initial energy with a relative precision that decreases exponentially with the number of degrees of freedom of the system.

Another interesting case is when the driving only modifies the parameters of a subsystem. For a compound system with two noninteracting parts $H(x_{1},x_{2})=H_{1}(x_{1})+H_{2}(x_{2})$, the marginal probability density is


(19) $$\begin{array}{rl} \rho (x_{1}) & =\frac{1}{\omega_{1+2}(E)}\int_{\Gamma_{2}}dx_{2}\;\delta (E-H(x_{1},x_{2}))\\ & =\frac{\omega_{2}(E-H(x_{1}))}{\omega_{1+2}(E)}. \end{array}$$

This state is passive if $\omega_{2}(\cdot )$ is an increasing function, which is the case for systems with positive absolute temperature. Hence, if system 1 has been in contact with any other system, then it will always be in a passive state. Also, if the external agent can manipulate only some of the degrees of freedom of a system and the interaction between these degrees of freedom and the rest of the system is negligible, then the effective state available to the external agent is (19), which is passive.

## Conclusions

Let us summarize the novel aspects introduced in this Perspective regarding the objectivity of the arrow of time, entropy, and the second law:

1. The analysis of entropy and irreversibility based on Einstein’s observable-dependent entropy.
2. The role of cooling environments and symmetry breaking in the origin of low-entropy states.
3. The increase of entropy in copying information, when the information in the original and the copy fade out, being both decoupled.
4. The assessment of ergotropy of the microcanonical state of large systems.

The first point helped us understand that irreversibility is objective and independent of the scale. It essentially reflects the return of an observable to its likely values after an initial, low-entropy fluctuation. Using observable-dependent entropy, we argued that the entropy of a system and the notion of equilibrium are subjective concepts, whereas temperature is objective.

The second contribution reveals a time-asymmetric origin of giant fluctuations. It allows us to replace the past hypothesis—a specific low-entropy initial state of the universe—with the milder assumption of an expanding universe whose background radiation temperature decreases over time.

The third contribution provides a firmer foundation for the coincidence between the thermodynamic arrow of time and traces or footprints.

Finally, the fourth point represents a new attempt to uncover the mechanical rationale behind the Kelvin–Planck statement of the second law of thermodynamics.

As mentioned in the Introduction section, this Perspective does not aim to formulate a consistent and objective derivation of thermodynamics within Newtonian mechanics. It does not even provide definitive answers to all issues concerning the objectivity of irreversibility, entropy, and the second law. Instead, it seeks to revive the problem of the objectivity of the arrow of time and the second law, revisiting parts of the relevant literature, and introducing new insights inspired by recent advances in statistical mechanics, stochastic thermodynamics, and the thermodynamics of information. The ultimate goal is to encourage further discussion that incorporates these ideas and addresses the examples introduced in the article.

## Supplementary Material

pgag288_Supplementary_Data

## Notes

a Note that the sets $\gamma_{E}$ and $\Gamma (A)\cap \gamma_{E}$ are 2M−1-dimensional manifolds. Consequently, they have zero 2M-dimensional volume. ω(A) is defined as the (2M−1)-dimensional volume of the set $\Gamma (A)\cap \gamma_{E}$ with respect to the hypersurface measure induced on $\gamma_{E}$ by the Euclidean metric on the phase space *Γ*. See next section and footnote b for a more detailed discussion in the context of observable-dependent entropy.

b Notice that $\omega_{A}(a;E)$ and ω(E) have different dimensions. The ratio (3) has dimensions $A^{-1}$ as expected for a probability density. Expressions (1) and (3) apply to continuous observables. One can also consider a discrete observable by replacing the Dirac delta δ(a−A(X)) in (1) with a Kronecker delta. In this case, $\omega_{A}(a;E)$ and ω(E) have the same dimensions and $\rho_{A}(x)$ in (3) becomes a dimensionless probability.

c Another line of research explores *autonomous demons* (37, 38). These are autonomous bipartite systems subject to nonequilibrium constraints, such as temperature or chemical potential gradients, that drive them into a nonequilibrium steady state. In some cases, this regime can be interpreted as an information engine in which one subsystem acts as a demon and the other as a working substance. The reconciliation with the second law in this case is straightforward: although there is an information flow between the two subsystems, the operation of the device is sustained by the steady entropy production associated with the nonequilibrium constraints (37, 39).
