# Supplementary material for: Is entropy objective? Rethinking the foundations of irreversibility and the second law
Source: PNAS Nexus. 2026 Aug 26;5(9):pgag288. doi: 10.1093/pnasnexus/pgag288 (PMC13560077; doi:10.1093/pnasnexus/pgag288)
Supplement: Supplementary file 1 [file pgag288_supplementary_data.pdf]

PNAS Nexus, YEAR, pp. 1–3

doi: DOI HERE
Advance Access Publication Date: Day Month Year
Supplementary Material:

SUPPLEMENTARY MATERIAL:

Is Entropy Objective? Rethinking the Foundations of Irreversibility and the Second Law

Juan M.R. Parrondo[a,*]

[a]Departamento de Estructura de la Materia, Física Térmica y Electrónica and GISC, Universidad Complutense de Madrid, 28040, Madrid, Spain
[*]To whom correspondence should be addressed: parrondo@ucm.es
FOR PUBLISHER ONLY Received on Date Month Year; accepted on Date Month Year

Abstract

Supplementary Material of *Is Entropy Objective? Rethinking the Foundations of Irreversibility and the Second Law.*

1. Objectivity of temperature

The derivative of entropy with respect to energy yields different values depending on which other quantities are kept constant [1]. For instance, for an ideal gas:

$$\left(\frac{\partial S}{\partial E}\right)_{V,N} = \frac{1}{T}; \qquad \left(\frac{\partial S}{\partial E}\right)_{P,N} = \frac{5}{3\,T}. \tag{1}$$

These are mathematical identities suggesting that the definition of temperature (10) in the main text could yield different results depending on the choice of observables in addition to the system energy.

The following example clarifies this issue and shows that the definition of temperature in the main text is objective and coincides with the standard thermodynamic temperature, as long as one can distinguish in an isolated system two parts, A and B, with well-defined energies E_A and E_B, and the entropy associated with E_A or E_B is additive.

Two gases, 1 and 2, exchange energy through a rigid wall, and gas 1 is subjected to a constant pressure P. The situation is easier to analyze if we imagine a piston of mass M in a uniform gravitational field g, as shown in Fig. 1. Then the pressure is $P = Mg/a$, where a is the piston's area, equal to the base area of the container. One can conceive different entropies depending on the choice of observables for describing the global system. For instance: a) the entropy $S_{E_1}(\epsilon; E)$ associated with the energy of gas 1; b) the entropy $S_{E_A}(\epsilon; E)$ associated with the energy $E_A = E_1 + Mgz = E_1 + PV_1$ of the subsystem formed by gas 1 plus the piston; and c) the entropy $S_{E_1,Z}(\epsilon, z)$ associated with the energy of gas 1 and the height of the piston Z.

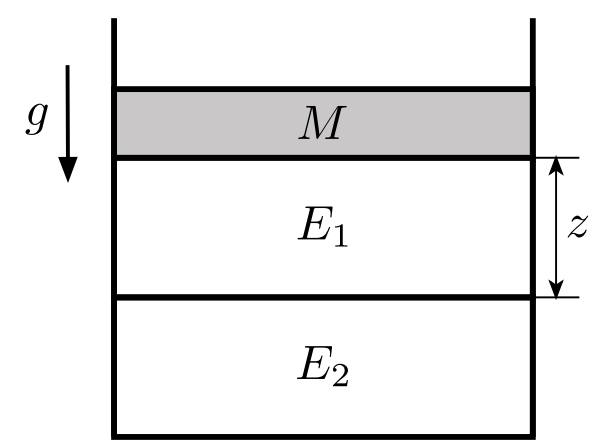

Fig. 1. Two gases, 1 and 2, exchange energy, and the upper one is subjected to a constant pressure. The pressure $P = Mg/a$ is exerted by a piston of area a, mass M, and weight Mg, g being the gravitational acceleration.

To calculate these entropies, we use the expression for the volume of the energy shell of an ideal gas with N molecules in a volume V and with total energy E, which is

$$\omega(E, V) = C_N E^{3N/2} V^N \tag{2}$$

where C_N only depends on the number of particles. In the following, C and C' denote any multiplicative constant that contributes only an additive constant to the entropy and does not affect the behavior of the observables of interest.

Applying this expression to the states compatible with $E_1 = \epsilon$ and total energy E, we get:

$$\begin{aligned}\omega_{E_1}(\epsilon; E) &= C \int_0^{(E-\epsilon)/(Mg)} dz\, \epsilon^{3N_1/2} z^{N_1} (E - Mgz - \epsilon)^{3N_2/2} \\ &= C'\, \epsilon^{3N_1/2} (E-\epsilon)^{3N_2/2+N_1+1} \int_0^1 dx\, x^{N_1} (1-x)^{3N_2/2}\end{aligned} \tag{3}$$

© The Authors. All rights reserved.

where we have used the change of variable $x = Mgz/(E-\epsilon)$. For $S_{E_A}(\epsilon;E)$, we have

$$\begin{aligned}\omega_{E_A}(\epsilon;E) &= C\int_0^{\epsilon/(Mg)} dz\,(\epsilon - Mgz)^{3N_1/2} z^{N_1}(E-\epsilon)^{3N_2/2}\\ &= C'\,\epsilon^{5N_1/2+1}(E-\epsilon)^{3N_2/2}\int_0^1 dx\, x^{N_1}(1-x)^{3N_2/2}\end{aligned} \tag{4}$$

where we have used the change of variable $x = Mgz/\epsilon$.

In the third case, $S_{E_1,Z}(\epsilon,z;E)$, we have

$$\omega_{E_1,Z}(\epsilon,z;E) = C\,\epsilon^{3N_1/2} z^{N_1}(E-\epsilon-Mgz)^{3N_2/2} \tag{5}$$

Then, we get the three entropies associated with each choice of observables, for $N_1, N_2 \gg 1$ and up to an additive constant:

$$S_{E_1}(\epsilon;E) = \frac{3N_1}{2}\,k\ln\epsilon + \frac{2N_1+3N_2}{2}\,k\ln(E-\epsilon) \tag{6}$$

$$S_{E_A}(\epsilon;E) = \frac{5N_1}{2}\,k\ln\epsilon + \frac{3N_2}{2}\,k\ln(E-\epsilon). \tag{7}$$

$$S_{E_1,Z}(\epsilon,z;E) = \frac{3N_1}{2}\,k\ln\epsilon + \frac{3N_2}{2}\,k\ln(E-\epsilon-Mgz) + N_1\ln z \tag{8}$$

Let us analyze each of these entropies and whether they can be used to define a temperature. Consider the derivative of the entropies with respect to the relevant energy:

$$\frac{S_{E_1}(\epsilon;E)}{\partial\epsilon} = \frac{3N_1k}{2\epsilon} - \frac{(2N_1+3N_2)k}{2(E-\epsilon)} \tag{9}$$

$$\frac{\partial S_{E_A}(\epsilon;E)}{\partial\epsilon} = \frac{5N_1k}{2\epsilon} - \frac{3N_2k}{2(E-\epsilon)} \tag{10}$$

$$\frac{\partial S_{E_1,Z}(\epsilon,z;E)}{\partial\epsilon} = \frac{3N_1k}{2\epsilon} - \frac{3N_2k}{2(E-\epsilon-Mgz)} \tag{11}$$

In expression (10), the additivity of entropy is apparent: we can identify the first term as the entropy of gas 1, and the second term as the entropy of gas 2. Then, the temperature of gas 1 is given by the first term in (10):

$$\frac{1}{T_1} = \frac{5N_1k}{2\epsilon} \tag{12}$$

This expression is analogous to a derivative of the entropy at constant pressure, i.e., the right equation in (1). Although the factor is 5/2, Eq. (12) yields the correct value of the temperature at equilibrium, because the equilibrium value of E_A is $\epsilon_A^{\rm eq} = \epsilon_1^{\rm eq} + Mgz^{\rm eq} = 3N_1kT_1/2 + PV_1 = 5N_1kT_1/2$.

In the third case $S_{E_1,Z}(\epsilon,z;E)$, we can split the entropy into three terms: the first one, $S^{(1)}_{E_1,Z}(\epsilon,z;E) \equiv (3N_1k/2)\ln\epsilon$ corresponding to gas 1, the second term corresponding to gas 2, and the third term corresponding to the piston. Then, the temperature of gas 1 is:

$$\frac{1}{T_1} = \frac{\partial S^{(1)}_{E_1,Z}(\epsilon,z;E)}{\partial\epsilon} = \frac{3N_1k}{2\epsilon}. \tag{13}$$

This choice of observables coincides with the definition of temperature using the derivative of the entropy at constant volume, i.e. the left expression in (1). Notice that both Eq. (12) and Eq. (13), yield the same result if the energy, either E_1 or E_A, is correctly identified.

Finally, in (6), one cannot identify terms that only depend on properties of each gas, A or B, and it is not possible to split the total entropy into two contributions from each gas.

The example illustrates that temperature can be objectively defined whenever we can identify two parts in a global system and the total entropy associated with the energy of one of the parts is additive, i.e., can be split into two contributions, each corresponding to one part of the system.

2. Thermodynamic constraints to copying and erasing information

The discussion of traces and registers in section 10 of the main text can be illustrated using a simple model of a single-domain ferromagnetic material, namely, the globally coupled Ising model. To analyze the copy and subsequent erasure of information, let us consider two coupled Ising models, A and B, with N spins $s_i^{(A)} = \pm 1$ and $s_i^{(B)} = \pm 1$, $i = 1,\dots,N$, and Hamiltonian

$$\begin{aligned}H &= -\frac{1}{N}\sum_{j>i}^{N}\left[J_A s_i^{(A)}s_j^{(A)} + J_B s_i^{(B)}s_j^{(B)}\right] - \frac{J_{AB}}{N}\sum_{i,j=1}^{N} s_i^{(A)}s_j^{(B)}\\ &= -\frac{N}{2}\left[J_A m_A^2 + J_B m_B^2 - \frac{J_A+J_B}{N} + 2J_{AB}m_Am_B\right]\end{aligned} \tag{14}$$

where $m_\alpha \equiv \sum_i s_i^{(\alpha)}/N$, with $\alpha = A, B$, is the magnetization per spin in each system. The Hamiltonian depends on three parameters: the coupling J_α between the spins of each system and the coupling J_{AB} between the two systems. Notice that the magnetization m_A of system A acts as an external field for system B, and vice versa.

If $J_{AB} = 0$, the two systems are decoupled and their behavior in contact with a thermal bath at temperature T is well known [2]. The probability distribution of the magnetization m_α is proportional to $\exp[-F_\alpha(m_\alpha)/(kT)]$, where the free energy is given by

$$F_\alpha(m) = N\left[-\frac{J_\alpha m^2}{2} - kTh\left(\frac{m+1}{2}\right)\right] \tag{15}$$

where

$$h(p) \equiv -p\ln p - (1-p)\ln(1-p) \tag{16}$$

is the binary entropy function. The free energy has one minimum at $m = 0$ if $J_\alpha \le J_{\rm crit} = 2kT$ (paramagnetic phase), and two minima if J_α is above this critical value $J_{\rm crit}$ (ferromagnetic phase). In the latter case, a symmetry breaking occurs, and the system gets trapped in configurations with positive or negative magnetization. Well above $J_{\rm crit}$, the probability that the magnetization changes sign decreases exponentially with N. When increasing J_α from the paramagnetic to the ferromagnetic phase, the system randomly adopts a positive or negative magnetization. Hence, the Ising model provides a natural framework to study the creation of random macroscopic bits, as well as copying [3].

The creation and copying of information can be implemented through the following protocol, starting from $J_{AB} = J_A = J_B = 0$. We first induce a symmetry breaking in system A, by driving J_A from 0 to $J_{\rm max} > J_{\rm crit}$ above the critical value of the coupling. The system randomly adopts one of the two possible informational states with positive or negative average magnetization $\langle m_A\rangle$. If we couple the two systems by shifting J_{AB} from 0 to $J_{\rm max}$, system B adopts a magnetization $\langle m_B\rangle$, with the same sign as $\langle m_A\rangle$, that is, we have copied the magnetization sign from system A to B. We can simultaneously drive system B to the ferromagnetic phase, $J_B : 0 \to J_{\rm max}$ if we want to keep the copy when the two systems are decoupled.

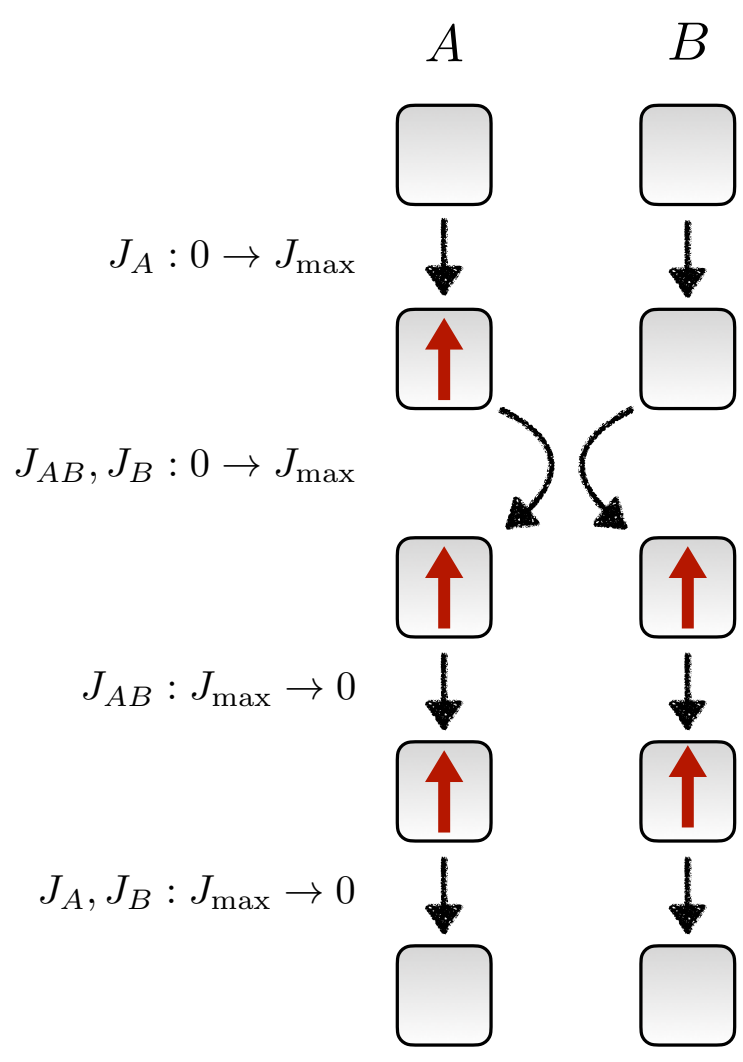

Fig. 2. Copying and erasing with two single-domain magnets.

We now analyze two different ways of erasing the original bit $s_A \equiv \operatorname{sgn}\langle m_A\rangle$ and the copy $s_B \equiv \operatorname{sgn}\langle m_B\rangle$. One can erase the two bits after decoupling the two systems, following the protocol: $J_{AB} : J_{\max} \to 0$, $J_A : J_{\max} \to 0$, and $J_B : J_{\max} \to 0$, as in Fig. 2. This process is irreversible. To see this, consider the time-reversal protocol. It first consists of a spontaneous symmetry breaking in each system. The probability that the systems adopt opposite magnetizations is 1/2. If this happens, when we couple the two systems by increasing J_{AB}, an irreversible hysteretic relaxation flips one of the two magnetizations to match the sign of $\langle m_A\rangle$ and $\langle m_B\rangle$.

It turns out that this irreversibility manifests as heat dissipation in the forward process. In [4, 5], we have derived the following relationship between irreversibility and entropy production in an arbitrary process starting at thermal equilibrium at temperature T:

$$W - \Delta F \geq kTD\left[\rho_{\mathrm{F}}(x)||\rho_{\mathrm{B}}(x)\right]. \tag{17}$$

Here W is the work done by the external agent that modifies parameters in the system Hamiltonian, ΔF is the increment in equilibrium free energy during the process, and k is the Boltzmann constant. x denotes an arbitrary observable (or set of observables), evaluated at one or multiple times during the process. $\rho_{\mathrm{F}}(x)$ is the probability distribution of x in the process, whereas $\rho_{\mathrm{B}}(x)$ is the probability distribution of the same observables when the protocol is run backward. Finally, $D(\rho||\sigma)$ is the Kullback-Leibler divergence between two probability distributions defined as

$$D\left[\rho(x)||\sigma(x)\right] \equiv \int dx\, \rho(x) \ln \frac{\rho(x)}{\sigma(x)}. \tag{18}$$

If x is a discrete variable, then the integral is replaced by a sum. Moreover, the inequality (17) is met if x is the only variable out of equilibrium in the forward and backward processes [6].

Let us apply this result to the two magnets setting x equal to the sign of the magnetization of the two systems $x = (s_A, s_B)$ right before the erasure, i.e., before J_A and J_B are decreased down to zero in the last step of the forward process. The probability distributions are

$$\rho_{\mathrm{F}}(s_A, s_B) = \frac{1}{2}\,\delta_{s_A,s_B} \tag{19}$$

$$\rho_{\mathrm{B}}(s_A, s_B) = \frac{1}{4} \tag{20}$$

$$\tag{21}$$

for all combinations with $s_A = s_B = \pm 1$. Since the process is a cycle $\Delta F = 0$ and (17) yields

$$W \geq kT\left[\frac{1}{2}\ln\frac{1/2}{1/4} + \frac{1}{2}\ln\frac{1/2}{1/4}\right] = kT\ln 2. \tag{22}$$

The work is dissipated as heat into the thermal bath at temperature T. Then, the entropy increases by $\Delta S = W/T \geq k\ln 2$. Here, we refer to the entropy associated with the magnetization of the two systems and the energy of the bath.

Eq. (17) is valid for any physical system [5]. Hence, these results apply to any pair of systems capable of creating, copying, and erasing information, regardless of their physical nature. Examples include Brownian particles in double-well potentials, molecular isomers, and single-electron boxes.

The inequality in (22) can be met in the case of Ising models if the protocol is quasistatic, as shown in [3], where the role of symmetry breaking in the energetics of these information processes is discussed in detail.

On the other hand, if we swap the steps of this protocol and erase the first bit when the two systems are still coupled: $J_A : J_{\max} \to 0$, $J_{AB} : J_{\max} \to 0$, and $J_B : J_{\max} \to 0$, then the resulting cycle is fully reversible and can be completed without heat dissipation and zero entropy production. The difference in the energetics of these two copying-erasure processes can also be explained by the differing number of critical points crossed in the final part of each process. In the irreversible erasure, each system crosses its critical point, whereas in the reversible erasure, the critical point of system A is avoided because J_A is driven from $J_{\max}$ to zero when the two systems are still coupled [3].

References

1. H.B. Callen. *Thermodynamics and an introduction to thermostatistics, 2nd edition.* John Wiley and Sons, New York, 1985.
2. P. M. Chaikin and T. C. Lubensky. *Principles of Condensed Matter Physics.* Cambridge University Press, 1995.
3. J.M.R. Parrondo. The Szilárd engine revisited: Entropy, macroscopic randomness, and symmetry breaking phase transitions. *Chaos: An Interdisciplinary Journal of Nonlinear Science*, 11(3):725–733, September 2001.
4. R. Kawai, J.M.R. Parrondo, and C. Van den Broeck. Dissipation: The phase-space perspective. *Physical Review Letters*, 98(8):080602, 2007.
5. J.M.R. Parrondo, C. Van den Broeck, and R. Kawai. Entropy production and the arrow of time. *New Journal of Physics*, 11(7):073008, jul 2009.
6. A. Gomez-Marin, J.M.R. Parrondo, and C. Van den Broeck. The footprints of irreversibility. *Europhysics Letters*, 82(5):50002, may 2008.
